# Modulation of Morphine Analgesia, Antinociceptive Tolerance, and Mu-Opioid Receptor Binding by the Cannabinoid CB2 Receptor Agonist O-1966

**DOI:** 10.3389/fphar.2022.803331

**Published:** 2022-04-21

**Authors:** Zachary W. Reichenbach, Kelly DiMattio, Suren Rajakaruna, David Ambrose, William D. Cornwell, Ronald J. Tallarida, Thomas Rogers, Lee-Yuan Liu-Chen, Ronald F. Tuma, Sara Jane Ward

**Affiliations:** ^1^ Center for Substance Abuse Research (CSAR), Department of Neural Sciences, Lewis Katz School of Medicine, Temple University, Philadelphia, PA, United States; ^2^ Department of Gastroenterology and Hepatology, Temple University Hospital, Philadelphia, PA, United States; ^3^ Center for Inflammation, Translational, and Clinical Lung Research, Lewis Katz School of Medicine, Temple University, Philadelphia, PA, United States

**Keywords:** morphine, CB2 receptor agonist, antinociception, antinociceptive tolerance, inflammation

## Abstract

Acutely, non-selective cannabinoid (CB) agonists have been shown to increase morphine antinociceptive effects, and we and others have also demonstrated that non-selective CB agonists attenuate morphine antinociceptive tolerance. Activation of cannabinoid CB2 receptors reverses allodynia and hyperalgesia in models of chronic pain, and co-administration of morphine with CB2 receptor selective agonists has been shown to be synergistic. CB2 receptor activation has also been shown to reduce morphine-induced hyperalgesia in rodents, an effect attributed to CB2 receptor modulation of inflammation. In the present set of experiments, we tested both the acute and chronic interactions between morphine and the CB2 receptor selective agonist O-1966 treatments on antinociception and antinociceptive tolerance in C57Bl6 mice. Co-administration of morphine and O-1966 was tested under three dosing regimens: simultaneous administration, morphine pre-treated with O-1966, and O-1966 pre-treated with morphine. The effects of O-1966 on mu-opioid receptor binding were determined using [3H]DAMGO and [^35^S]GTPγS binding assays, and these interactions were further examined by FRET analysis linked to flow cytometry. Results yielded surprising evidence of interactions between the CB2 receptor selective agonist O-1966 and morphine that were dependent upon the order of administration. When O-1966 was administered prior to or simultaneous with morphine, morphine antinociception was attenuated and antinociceptive tolerance was exacerbated. When O-1966 was administered following morphine, morphine antinociception was not affected and antinociceptive tolerance was attenuated. The [^35^S]GTPγS results suggest that O-1966 interrupts functional activity of morphine at the mu-opioid receptor, leading to decreased potency of morphine to produce acute thermal antinociceptive effects and potentiation of morphine antinociceptive tolerance. However, O-1966 administered after morphine blocked morphine hyperalgesia and led to an attenuation of morphine tolerance, perhaps due to well-documented anti-inflammatory effects of CB2 receptor agonism.

## Introduction

Cannabinoid receptor agonists produce antinociception in a variety of animal models, and the majority of these effects appear to be mediated by CB1 receptors. Interactions between cannabinoid and opioid receptor systems remain an area of intense research, especially in light of the mounting importance of identifying safer and more effective pain therapies that may be able to reduce opioid use and associated harms. Acutely, the non-selective CB agonists Δ9-tetrahydrocannabinol (THC) and CP-55,940 have been shown to increase morphine antinociceptive effects (Smith et al., 1998; Manzanares et al., 1999; Finn et al., 2004; Tham et al., 2005; Vigano et al., 2005; Maguire and France 2018). We and others have also demonstrated that non-selective CB agonists attenuate morphine antinociceptive tolerance (Cichewicz et al., 2001; Cichewicz and Welch 2003; Fischer et al., 2010). The CB1 receptor is abundantly expressed throughout the central nervous system and identified as the cannabinoid receptor responsible for the “psychoactive” effects of non-selective cannabinoid agonists such as THC; therefore, it is presumed that these CB agonist effects on morphine tolerance are associated with their actions on the CB1 receptor. However, this remains to be demonstrated empirically.

Relative to CB1 receptors, detection of CB2 receptors in the CNS of naïve animals remains relatively low to absent, and by and large CB2 receptor activation does not lead to the range of CNS effects associated with CB1 receptor activation, such as euphoria, changes in mood, and alterations in cognition. However, CB2 receptor expression is upregulated within the CNS in animal models of chronic inflammatory or neuropathic pain (Zhang et al., 2003; Wotherspoon et al., 2005; Beltramo et al., 2006), and activation of CB2 receptors reverses allodynia and hyperalgesia in these models (Guindon and Hohmann 2008; Rahn et al., 2011). In addition, co-administration of morphine with CB2 receptor selective agonists synergistically inhibits inflammatory, post-operative and neuropathic pain in rodent models (Grenald et al., 2017; Yuill et al., 2017; Iyer et al., 2020) and reduces morphine-induced thermal hyperalgesia in rats (Tumati et al., 2012). While a preponderance of studies has demonstrated that tolerance is associated with a significant reduction in functional surface *µ* opioid receptors (Williams et al., 2013). Other studies have suggested that morphine tolerance is due at least in part to direct microglial activation and the release of proinflammatory cytokines (Hutchinson et al., 2007, see Hutchinson et al., 2011 for review). Our laboratory has extensively characterized the protective and anti-inflammatory effects of the CB2 receptor agonist O-1966 in several rodent models of CNS injury (Zhang et al., 2007; Adhikary et al., 2011; Elliott et al., 2011; Amenta et al., 2012; Ramirez et al., 2012; Ronca et al., 2015). As CB2 receptor activation has been shown to significantly modulate inflammatory responses, including inhibition of microglial activation, we hypothesized that CB2 receptor activation may lead to attenuation of morphine antinociceptive tolerance.

In the present set of experiments, we tested both the acute and chronic interactions between morphine and O-1966 treatments alone and in combination on antinociception and antinociceptive tolerance and hyperalgesia in C57Bl6 mice using a standard hot plate assay. Based on previous research, we hypothesized that O-1966 would be devoid of acute antinociceptive effects but would attenuate morphine antinociceptive tolerance. Because our first results from our acute hotplate experiments revealed an unpredicted attenuating effect of O-1966 on acute morphine antinociception, we proceeded in these acute studies as well as the tolerance studies to test administration of morphine and O-1966 under three dosing regimens: concurrent administration, morphine pre-treated with O-1966, and O-1966 pre-treated with morphine. Based on the results of these experiments revealing that the order of drug administration had dramatic effects on how these two drugs affected morphine analgesia and analgesic tolerance, we further tested the hypothesis that select interactive effects between O-1966 and morphine were a result of direct effects of this CB2 receptor agonist on the µ opioid receptor. The effects of O-1966 on mu-opioid receptor binding were determined using [3H]DAMGO and [^35^S]GTPγS binding assays. Lastly, as our behavioral data revealed that O-1966 could attenuate morphine antinociception but also facilitate morphine tolerance, we tested the hypothesis that O-1966 was interfering with mu-opioid receptor homodimerizationvia FRET analysis linked to flow cytometry.

## Materials and Methods

### Drugs

For *in vivo* experiments, O-1966 (Organix Laboratories, Massachusetts, USA) and SR144528 (RTI) were prepared in ethanol:Cremophor:Saline (1:1:18). Morphine was dissolved in 0.9% saline. All injections were given i. p. in a volume of 10 ml/kg. For *in vitro* experiments, O-1966 and SR144528 were dissolved in DMSO (final concentration 2% in assays) and morphine was dissolved in Milli-Q water. The affinity of O-1966 for CB1 and CB2 cannabinoid receptors was reported previously to be 5055 ± 984 and 23 ± 2.1 nmol/L, respectively (Wiley et al., 2002).

### Animals

All experiments were conducted in 7 to 8-week-old male C57BL/6 mice weighing 18–23 g (Taconic Laboratories, New York, USA). Studies were conducted in accordance with the guidelines approved by the Institutional Animal Care and Use Committee at Temple University. Animals were housed under a 12 h light/dark cycle with lights on at 07:00 h and maintained on a regular chow diet and had access to food and water *ad libitum* throughout the study. All experimental groups were n = 8/treatment condition.

### Measurement of Hot Plate Withdrawal Latency

Nociception was analyzed by means of a hot plate analgesia meter (Columbus Instruments, Columbus, OH). Mice were placed on a hot plate maintained at 54.0 ± 0.5°C. The latency to hind paw lick, hind paw lift, hind paw flutter, mouse shuffle, or mouse jump was measured to the nearest 0.1 s as described in Fischer et al., 2010. A maximal cutoff of 30 s was utilized to prevent injury to the paw tissue. Immediately after the end of the trial, mice were returned to their home cage. The latency to respond at 54°C was measured twice at 2 and 1.5 h prior to the beginning of drug administration, and these data were averaged to yield one baseline value. Following baseline latency measurements, multiple 30 min cycles were run and drugs and drug mixtures were administered cumulatively. During this procedure, cumulative doses of morphine, O-1966, or their combination were administered during the first min of each cycle (i.e., 30-min inter-injection interval), increasing in one-half log unit increments, and antinociceptive measurements were determined during the last minute of each cycle. Latencies obtained following drug administration were reported as Percent Maximal Possible Effect (%MPE). The following formula was utilized to calculate such:

$$\%MPE=\frac{\left(Experimental\;Latency-Average\;Baseline\;Latency\right)}{\left(Maximal\;Cut\;Off\;Time-Average\;Basline\;Latency\right)}\times 100$$

The antinociceptive effects of 1) morphine alone, 2) O-1966 alone, 3) their simultaneous administration, and 4) their simultaneous administration following CB2 antagonist treatment, were assessed in the same group of mice, with a 1 week washout period separating each drug or drug combination testing. In a separate group of mice, the antinociceptive effects of 1) morphine alone, 2) O-1966 administration followed 15 min later by morphine administration, and 3) morphine administration followed 15 min later by O-1966 administration were assessed with a 1-week washout period separating each drug or drug combination testing.

### Induction of Morphine Antinociceptive Tolerance

One day following assessment of hot plate withdrawal latencies and the generation of baseline morphine dose-response curves, separate groups of mice were treated twice daily separated by 10 h for 5 days, as described in Fischer et al., 2010, with two vehicle regimens (saline, cremophor vehicle), two morphine alone dosing regimens (32 mg/kg, 100 mg/kg), and three morphine + O-1966 dosing regimens (simultaneous, O-1966 followed 15 min by morphine, morphine followed 15 min by O-1966). Reassessment of hot plate withdrawal latencies and morphine dose response curves began 14 h after the last tolerance regimen injections.

### *In Vitro* Materials

[Tyrosyl-3, 5-^3^H(N)]-DAMGO (56 Ci/mmol) and [^35^S]GTPγS (1,250 Ci/mmol) were purchased from PerkinElmer Life Sciences (Boston, MA); sucrose, bovine serum albumin (BSA), phenylmethylsulfonyl fluoride, GDP and GTPγS were purchased from Sigma-Aldrich (St. Louis, MO). DMEM/F12, trypsin and penicillin/streptomycin were purchased from Gibco Life Technologies (Grand Island, NY). The following reagents were purchased from the indicated companies: geneticin (G418), Cellgro Mediatech, Inc. (Herndon, VA); EcoScint scintillation fluid, National Diagnostics (Atlanta, GA); fetal bovine serum (FBS), Atlanta Biologicals (Atlanta, GA). Naloxone and morphine were generously provided by the National Institute on Drug Abuse (Bethesda, MD).

### Cell Lines and Membrane Preparation

The following is a modified procedure from Wang et al. (Wang et al., 2005). CHO cells stably transfected with the rat mu-opioid receptor were established previously (Chen et al., 1995). Cells were cultured in 100-mm culture dishes in Dulbecco’s modified Eagle’s medium/F-12 HAM supplemented with 10% FBS, 0.3 mg/ml geneticin, 100 units/ml penicillin, and 100 g/ml streptomycin in a humidified atmosphere consisting of 5% CO2 and 95% air at 37 °C. Membranes were prepared according to a modified procedure of Zhu et al. (1997). Cells were washed twice and harvested in 1x PBS containing 0.5 mM EDTA and centrifuged at 500 *g* for 3 min. The cell pellet was suspended in lysis buffer (25 mM Tris, pH 7.4, 1 mM EDTA and 0.1 mM phenylmethylsulfonyl fluoride), passed through a 26 3/8-gauge needle 10 times and then centrifuged at 46,000 g for 30 min. The pellet was rinsed twice with lysis buffer and resuspended in 50 mM Tris-HCl buffer/0.32 M sucrose (pH 7.4), aliquoted and frozen in dry ice/ethanol, and stored at 80°C. All procedures were performed at 4 °C.

### Receptor Binding Assays

The binding affinity of O-1966 to rMOR was determined by competitive inhibition of [^3^H]DAMGO binding to CHO-rMOR membranes was performed with [^3^H]DAMGO at a concentration close to its K_d_ value (2 nM), using six concentrations (0.1 nM–1 μM) of unlabeled O-1966. The reaction was performed in 50 mM Tris-HCl buffer containing 1 nM EGTA and 0.1% (w/v) BSA (pH 7.4) at room temperature for 1 h in duplicate in a volume of 1 ml with 15–25 μg of membrane protein. Naloxone (10 μM) was used to define nonspecific binding. The reaction was terminated by filtration of bound and free [^3^H]DAMGO with GF/B filters presoaked with 50 mM Tris, pH 7.4, 0.1 mg/ml BSA, and 0.2% polyethyleneimine under reduced pressure. The filter was washed with ice-cold buffer containing 100 mM Tris (pH 7.6) and 0.154 M NaCl and radioactivity in filters were determined by liquid scintillation counting. This binding was repeated three times and data were analyzed and the K_i_ value of O-1966 was determined with GraphPad Prism Software.

### Ligand-Stimulated [35S]GTPγS Binding

To determine the effects of CB2 compounds on G protein activation at the mu-opioid receptor by morphine, we used clonal Chinese hamster ovary cells stably expressing the rat MOR (CHO-rMOR) due to their lack of endogenous cannabinoid receptors [^35^S]GTPγS binding was performed as previously described following a modified protocol (Zhu et al., 1997). Briefly, membranes (containing 10 µg protein) were incubated with 10 µM GDP and ∼0.4 nM [^35^S]GTPγS in reaction buffer (50 mM HEPES, 100 mM NaCl, 5 mM MgCl_2_, 1 mM EDTA) in the following two paradigms in a final volume of 0.5 ml:

#### Morphine pretreatment

0.5 µM morphine for 10 min at 30°C followed by 1 nM–10 µM CB2 compound (O-1966, SR144528, or O-1966 + SR144528).

#### CB2 pretreatment

1 nM–10 µM CB2 compound (O-1966, SR144528, or O-1966 + SR144528) for 10 min at 30°C followed by 0.5 µM morphine.

Reaction mixtures were incubated for 1 h at 30°C. Nonspecific binding was determined in the presence of 10 µM GTPγS. Subsequently, bound and free [^35^S]GTPγS were separated by filtration with GF/B filters under reduced pressure and the filter was washed with ice-cold buffer containing 50 mM Tris (pH 7.6), 5 mM MgCl_2_ and 50 mM NaCl. Radioactivity in filters was determined by liquid scintillation counting. All experiments were performed in duplicate and repeated three times. Data were analyzed and values were determined with GraphPad Prism Software.

### FRET Analysis

Fluorescence (Forsters) resonance energy transfer (FRET) analysis was used to determine the level of MOR dimerization by employing a modification of the flow cytometry method of Banning et al. (2010). The CHO cell line was transiently transfected with either rat MOR-CFP or MOR-YFP (molecular constructs a generous gift from Dr. Ping-Yee Law, University of Minnesota), or both to determine the energy transfer between MOR dimers. CHO cells were cultured in log phase and transfected with the 4D-Nucleofector (Lonza Group Ltd., Basel, Switzerland) using manufacturer’s procedure for this cell line. Cells were excited in the flow cytometer with a 405 nm laser, and the CFP emission was detected with a standard 450 nm filter, while the FRET was detected with a 530 nm filter. Control samples were established with non-transfected CHO cells, CHO cells transfected with either MOR-YFP or MOR-CFP alone, and cell mixtures of CHO-YPF (single transfection) and CHO-CFP (single transfection) cells. The degree of FRET is measured by the degree of fluorescence intensity in the FRET cytometry gate using mean fluorescence intensity. Flow cytometry was carried out with the Becton-Dickinson Influx cytometer (BD Biosciences, San Jose, CA).

### Data Analysis

The dose of morphine alone or O-1966 alone or in combination required to produce 50% maximum antinociceptive effect (ED50) during hotplate tests was derived using regression analysis (GraphPad Prism 5.0 software, Inc., La Jolla, CA).

when at least three data points were available on the linear portion of the dose-effect curve or by interpolation when only two data points (one above and one below 50%) were available. Acute studies were analyzed by comparing the expected effect with the observed effect using the principle of dose equivalence and application of a Student’s t-test. This approach was taken instead of dose addition and isobolographic analysis as it was determined that morphine produced a linear dose response curve while the dose response for O-1966 was hyperbolic (Tallarida and Raffa 2010).

**FIGURE 1:**
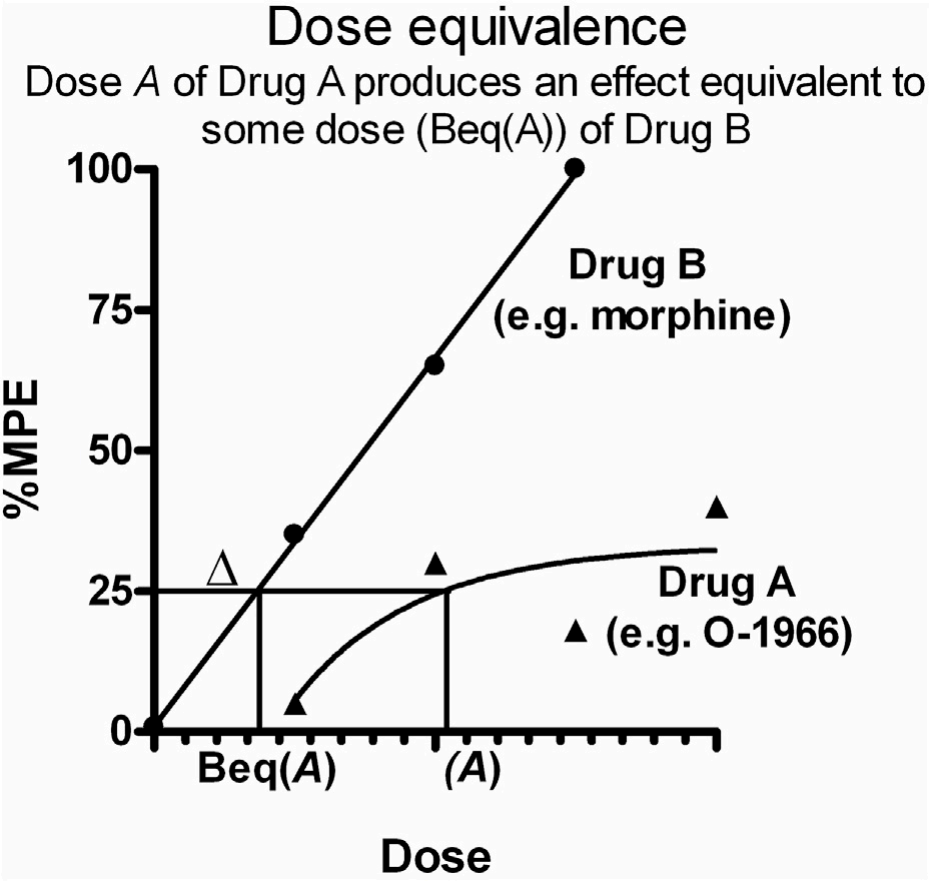
Graphical representation of application of dose equivalence analysis to data from two drugs producing dose-response effects fit to different slopes. Each dose A of Drug A (e.g. O-1966) is equally effective to some dose of a more efficacious drug (Drug B, e.g. morphine). This equi-effective dose of Drug B is designated Beq **(A)**, or Δ. In the drug combination **(A,B)**, the administered Drug B dose B is increased by Δ, and the sum of the two doses (B+ Δ) allows the calculation of expected effects. Analysis proceeds comparing the expected effect with the observed effect.

In dose equivalence analysis, the result of adding a given dose of Drug A (*a*) to a dose of Drug B (*b*) that produces a known effect level is predicted and then compared to the observed effect of the dose combination (*a, b*) (Figure 1). It is based on the principle that each dose *A* of Drug A (e.g. O-1966) is equally effective to some dose of a more efficacious drug (Drug B, e.g. morphine). As this equi-effective dose of Drug B is the equivalent dose in effect to dose *A,* it is designated *Beq (A),* or Δ. Therefore, in the combination (*A, B*), the administered Drug B dose *B* is increased by Δ, and the sum of the two doses (*B+* Δ) allows the calculation of expected effects. Analysis proceeds comparing the expected effect with the observed effect. Student’s *t*-test was used to compare the expected effect to the observed effect for all dose combinations in order to determine the nature of interaction between the morphine and O-1966 (additivity, sub-additivity, or synergy). For tolerance studies, doses producing a 50% reduction in nociception on the hotplate (ED50s) for morphine antinociception on Days 1 and 7 were calculated as the mean and SEM from individual animal ED50 calculations. Fold increases were determined by dividing Day 7 ED50s by Day 1 ED50s for each treatment group. Therefore, a relative potency of one suggests a lack of tolerance development (i.e. no shift in the morphine dose-effect curve). In contrast, a relative potency greater than one suggests that tolerance has developed (i.e. a rightward shift in the morphine dose-effect curve), and a quantitatively greater relative potency is indicative of increased tolerance development. Hyperalgesia was measured by comparing pre-drug baseline hotplate latencies between Day 1 and Day 7 using a Student’s t-test. GTPγS binding data were analyzed by two-way ANOVA with order and concentration as factors. Results of FRET analysis were evaluated with one-way ANOVA.

## Results

### Acute Morphine Antinociception

**FIGURE 2:**
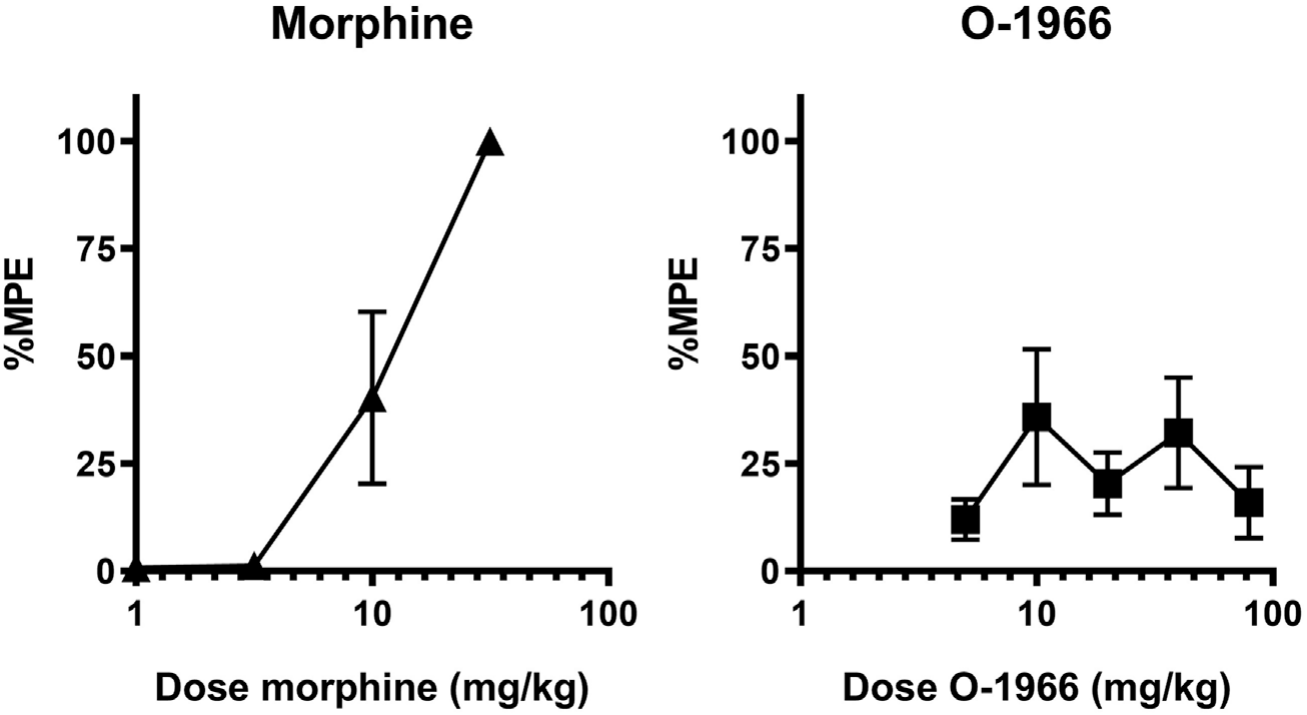
Effect of morphine and O-1966 alone on antinociception as measured by withdrawal latency on a 54°C hotplate. X-axis: Cumulative dose of morphine (A), or O-1966 (B) in mg/kg. *Y*-axis: antinociception as percent maximum possible effect. Each data point represents the mean (±S.E.M.) from eight mice.

Cumulative dosing of morphine produced dose-dependent antinociception that was linearly related to dose with an ED50 value of 9.1 (1.6) (Figure 2A). In contrast, the CB2 agonist O-1966 showed limited efficacy and values that fit to the standard hyperbolic dose-effect function using nonlinear regression (Figure 2B). The two fitted curves allowed for the determination of the expected additive effect for each dose combination tested for comparison with the experimentally derived (observed) effect (Table 1).

**TABLE 1:** Predicted additive and actual observed ED50 values for simultaneous administration of O-1966, SR144528, and morphine combinations on acute antinociception on the hotplate.

Dose: O-1966(+SR144528)+Morphine	Effect additive	Effect observed Simultaneous administration
1.25 + 3.0	25.1	9.77
2.5 + 10	47.4	17.2
5.0 + 30	88.1	18.2
10 + 100	100	70.4
20 + 300	100	78

**FIGURE 3:**
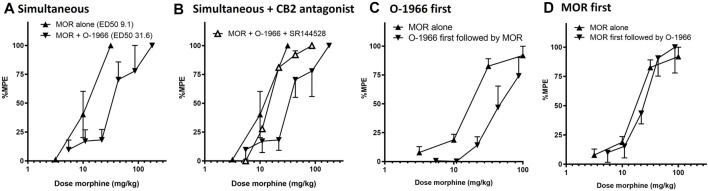
Order effect of morphine and O-1966 in combination on antinociception as measured by withdrawal latency on a 54°C hotplate. Morphine and O-1966 were either administered simultaneously **(A)**, simultaneous with SR144528 **(B)**, O-1966 15 min prior to morphine **(C)**, or morphine 15 min prior to O-1966 **(D)**. X-axis: Cumulative dose of morphine in mg/kg. *Y*-axis: antinociception as percent maximum possible effect. Each data point represents the mean (±S.E.M.) from eight mice. See Table 1 for doses of O-1966 and SR-144528 that corresponded to administered doses of morphine.

For the combination experiments, a dose of 2.5 mg/kg O-1966 was selected to be tested in combination with the approximate ED50 dose of 10 mg/kg morphine to generate rational dose combinations for the prediction and experimental determination of effect. We selected this dose of O-1966 based on previously demonstrated robust effects from our laboratory of O-1966 at the 5.0 mg/kg dose on neuroprotection in several models. A full range of O-1966 + morphine dose combinations were explored based on this ratio of equi-effective doses. The results showed that when administered at the same time, the combination of morphine and O-1966 was subadditive, with statistical analysis showing a significant difference (*p* < 0.05) between the observed effects and predicted additive effects (Figure 3A; Table 1). Pretreatment with CB2 antagonist at the same dose as CB2 agonist showed that SR144528 attenuated the sub-additive interaction and restored the morphine dose-effect curve. The ED50 (sem) was determined to be 31.5 (5.68) for morphine + O-1966, and 11.8 (1.97) for morphine + O-1966 + SR144528. Parallel line analysis (Tallarida and Murray 1987) was used to determine that the three lines have slopes that are not significantly different (Figure 3B; Table 1).

In a separate group of mice, it was also determined that the acute antinociceptive interaction between morphine and O-1966 was dependent on the order of administration prior to hotplate testing. Pretreatment with O-1966 15 min prior to morphine administration resulted in an approximate 2.5-fold shift in the morphine dose response curve (from an ED50 of 16.4 (1.1) to an ED50 of 43.8 (5.8) (Figure 3C), while no shift was observed when morphine was administered 15 min prior to O-1966 administration (ED50 20.8 (4.6)) (Figure 3D).

### Morphine Antinociceptive Tolerance and Hyperalgesia

**FIGURE 4:**
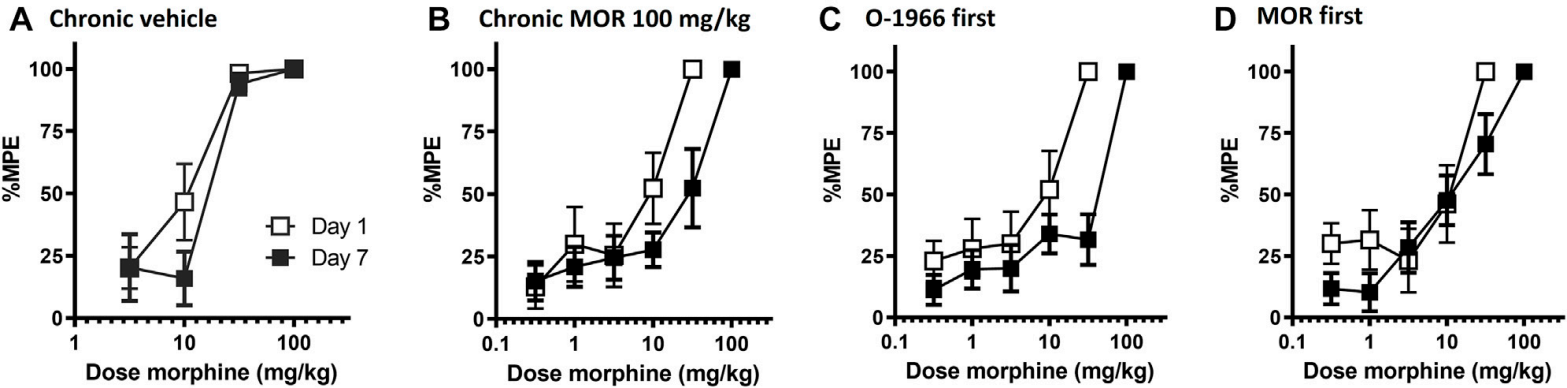
Effect of chronic dosing regimens of vehicle **(A)**, morphine **(B)**, O-1966 (data shown in Table 2), and morphine + O-1966 **(C,D)** on development of morphine antinociceptive tolerance. *X*-axis: Cumulative dose of morphine in mg/kg. *Y*-axis: antinociception as percent maximum possible effect. Each data point represents the mean (±S.E.M.) from eight mice. Open squares represent morphine antinociceptive effect on Day 1, and closed squared represent morphine antinociceptive effect in the same mice on Day 7 following a 5 day chronic dosing regimen. Titles above the graphs describe agents administered during the 5 day dosing regimen. In groups that received morphine and O-1966 during the dosing regimen, drugs were given 15 min apart.

Chronic administration for 5 days with either saline or cremophor vehicle had no effect on morphine antinociception. Chronic administration of morphine produced a dose-dependent rightward shift in the morphine dose response curve, with twice daily administration of 100 mg/kg morphine leading to an approximate 4-fold shift in morphine’s antinociceptive potency (Table 2; Figure 4A,B).

**TABLE 2:** Effect of chronic dosing regimens on development of morphine antinociceptive tolerance.

Tolerance regimen	Day 1 ED50(sem)	Day 7 ED50(sem)	Fold shift
Saline	7.2 (1.9)	4.1 (0.6)	0.6
Cremophor vehicle	8.0 (2.9	12.2 (2.3)	1.5
Morphine 32	4.0 (1.3)	8.6 (3.4)	2.2
Morphine 100	7.1 (2.0)	27.8 (10.8)	3.9
O-1966 then Morphine 32	7.5 (1.7)	15.6 (8.2)	2.1
O-1966 then Morphine 100	6.4 (2.3)	42.0 (16.5	6.6
Morphine 100 then)-1966	6.6 (1.8)	12.6 (3.3)	1.9
O-1996	10.7 (2.4)	9.9 (2.6)	0.9

Pretreatment with 5.0 mg/kg O-1966 15 min prior to each morphine injection during the tolerance regimen led to a further rightward shift in the morphine dose response curve, with twice daily administration of O-1966 + morphine leading to an approximate 6.5-fold shift in morphine’s antinociceptive potency (Table 2; Figure 4C). Conversely, when 5.0 mg/kg O-1966 was administered 15 min following each morphine injection during the tolerance regimen, the rightward shift in the morphine dose response curve was smaller than that seen following morphine alone treatment, producing an approximate 2-fold shift in morphine’s antinociceptive potency (Table 2; Figure 4D). Chronic administration for 5 days with O-1966 alone had no effect on morphine antinociception (Table 2).

### Morphine-Induced Hyperalgesia

**FIGURE 5:**
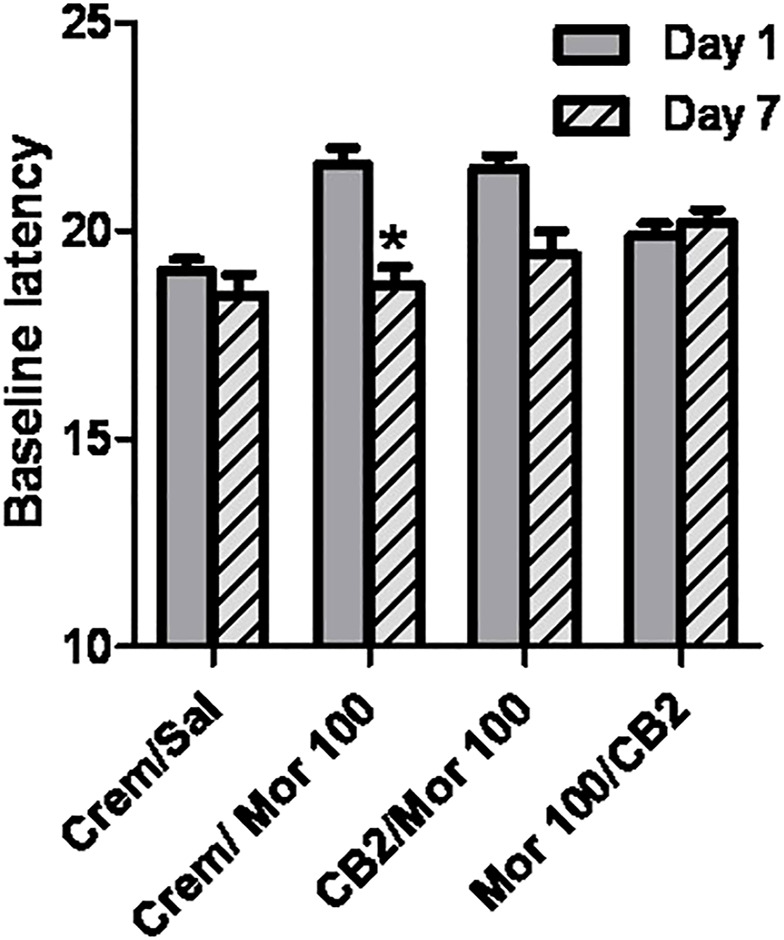
Effect of chronic dosing regimens on development of morphine hyperalgesia. *X*-axis: Agents administered during the 5 day chronic dosing regimen. *Y*-axis: Baseline latency to lift, lick, or shuffle hindpaw(s) on a 54°C hotplate prior to morphine antinociceptive testing. Each bar represents the mean (±S.E.M.) from eight mice. Solid grey bars represent baselines on Day 1, and hatched grey and black bars represent baselines in the same mice on Day 7 following a 5 day chronic dosing regimen. In groups that received morphine and O-1966 during the dosing regimen, drugs were given 15 min apart.

The presence of hyperalgesia was determined by comparing withdrawal latencies at baseline on day 1 with those measured on day 7 following the 5-day dosing regimen. The only group that showed a significant decrease in thermal sensitivity on day 7 as compared with day 1 was the group that received Morphine 100 mg/kg alone, as measured by Student’s t-test, *p* < 0.05 (Figure 5). No other treatment regimen produced a significant change in baseline sensitivity to the hotplate.

### Displacement of [3H]DAMGO by O-1966

**FIGURE 6:**
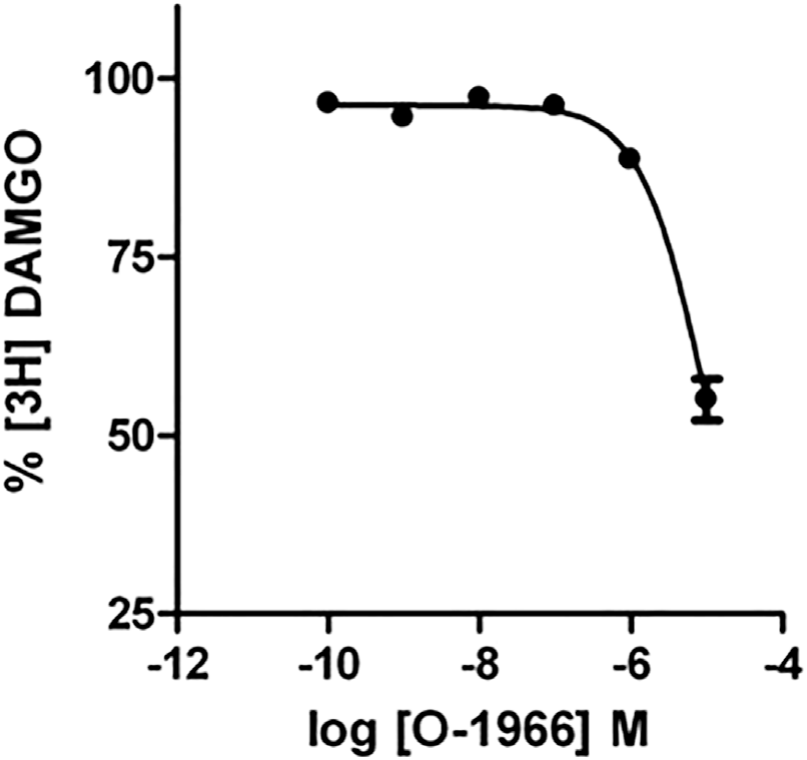
Effect of increasing concentrations of O-1966 on [^3^H] DAMGO binding. O-1966 has a low affinity for the rMOR. At a dose of 10 μM, O-1966 inhibited ∼50% of radiolabled [^3^H]DAMGO (2 nM) to rMOR. Lower doses (0.1 nM–1 µM) of O-1966 have no effect on [^3^H]DAMGO binding to CHO-rMOR. Each data point represents the mean (±S.E.M.) from three independent experiments run in duplicate.

Competition binding with O-1966 and [^3^H]DAMGO (2 nM) revealed that O-1966 does not have appreciable affinity for the CHO-rMOR. The K_i_ value for O-1966 was 3.04 µM (Figure 6).

### [35S]GTPγS Binding in CHO Cell Membranes

**FIGURE 7:**
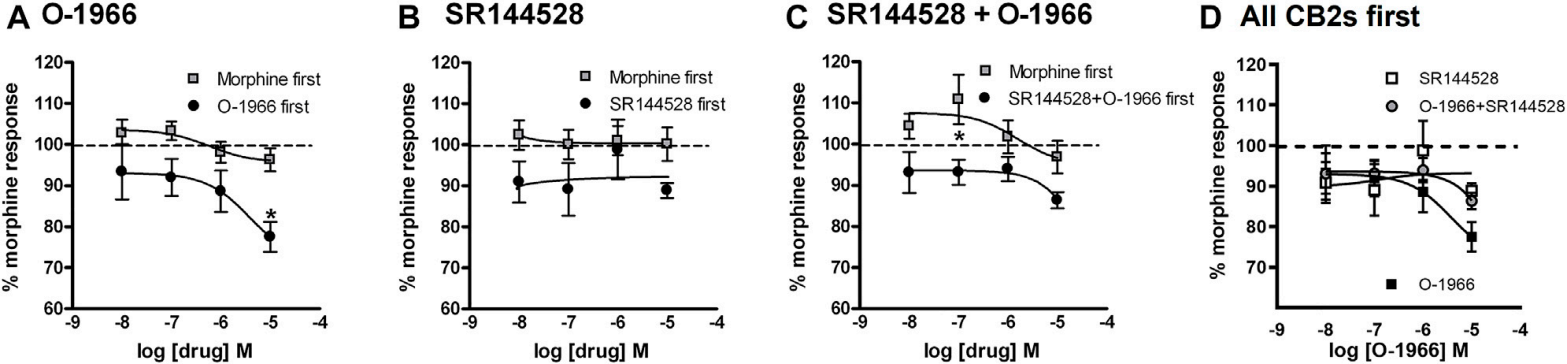
Effect of increasing doses of O-1966 and SR144528 on morphine-stimulated GTPγS binding. O-1966 given before, but not after, morphine inhibits morphine response in [^35^S]GTPγS binding assays. At the highest dose (10 µM) O-1966, there is a 20% reduction in the morphine response from baseline. Open squares indicate 10 min pretreatment with 0.5 µM morphine and closed squares indicate 10 min pretreatment with varying concentrations of either O-1966 **(A)**, SR144528 **(B)**, or a combination of SR144528 with O-1966 **(C)** (10 nM–10 µM). When the cannabinoid compounds are administered first, the co-administration of SR144528 and O-1966 indicates that the inhibitory effect of O-1966 can be blocked by SR144528 **(D)**. Each data point represents the mean (±S.E.M.) of three independent experiments run in duplicate (*) indicates a statistically significant (*p* < 0.05) difference from baseline morphine response.

In the O-1966 experiment (Figure 7A), two-way ANOVA revealed a significant effect of order of application [F (1,16) = 18.19, *p* < 0.05] and significant effect of O-1966 concentration [F (3, 16) = 3.253, *p* < 0.05 but no significant interaction [F (3, 16)<1, ns]. Bonferroni posttest revealed a significant difference between treatment groups at the 10 μM concentration of O-1966. In the SR144528 experiment (Figure 7B), two-way ANOVA revealed a significant effect of order of application [F (1,16) = 7.178, *p* < 0.05] but no significant effect of SR144528 concentration [F (3, 16)<1, ns] and no significant interaction [F (3, 16)<1, ns]. Bonferroni posttest revealed no significant difference between treatment groups at any concentration of SR144528. In the SR144528 + O-1966 experiment (Figure 7C), two-way ANOVA revealed a significant effect of order of application [F (1,16) = 17.97, *p* < 0.05] but no significant effect of SR144528 concentration [F (3, 16)<1, ns] and no significant interaction [F (3, 16)<1, ns]. Bonferroni posttest revealed a significant difference between treatment groups at the 0.1 μM concentration of SR144528 + 0.1 μM concentration of O-1966. A comparison of the effect of O-1966, SR144528, and SR144528+O-1966 pretreatments shows that O-1966 alone at the 10 μM concentration attenuates morphine-stimulated [35S]GTPγS binding, and that this attenuation is blocked by co-administration of SR144528 (Figure 7D).

### MOR Dimerization

Experiments were carried out using FRET analysis to determine the impact of O-1966 treatment on MOR dimers. CHO cells were co-transfected with molecular constructs which express MOR-CFP and MOR-YFP and assessing the energy between the CFP and YFP fluorescence partners. The results (Table 3) show that co-transfected cells treated with either morphine or O-1966, followed with either O-1966 or morphine, respectively, showed some reduction in the level of dimerization (based on inhibition of FRET). However, the data show that the pre-treatment with O-1966 did not significantly change the dimer status when compared to morphine pre-treatment. In no case was there a statistically significant difference between the morphine and O-1966 pretreatment groups.

**TABLE 3:** FRET Analysis of morphine and O-1966 co-treated cells.

Group	FRET inhibition
Control	0 ± 0
morphine (10 min)+O-1966 (60 min)	17.7 ± 4.2
O-1966 (10 min)+morphine (60 min)	11.9 ± 3.8
morphine (60 min) + O-1966 (10 min)	16.3 ± 4.2
O-1966 (60 min) + morphine (10 min)	18.6 ± 2.9
Morphine	5.7 ± 1.4
O-1966	−0.5 ± 3.7

## Discussion

The present results demonstrate modulation of morphine antinociception and antinociceptive tolerance by the CB2-selective agonist O-1966. Our results support the previous finding by Tumati et al. (2012) that CB2 receptor agonism attenuated the development of morphine hyperalgesia, and partially supported the findings by Zhang et al., 2016 and Lin et al., 2018 that co-administration of CB2 receptor agonists with morphine reduced development of antinociceptive tolerance in rodent models of cancer pain and chemotherapy-induced neuropathic pain respectively.

In contrast to our overall hypotheses, however, we observed that co-administration of the CB2-selective agonist attenuated acute morphine antinociception, while having more complex effects on the development of morphine tolerance, with all of these findings depending on the order of administration of O-1966 and morphine. The effect of O-1966 on acute morphine antinociception was dependent on O-1966 being administered prior to or simultaneous with morphine and was reversed by co-administration of the CB2 selective antagonist SR144528. In contrast, when morphine was administered prior to O-1966, O-1966 had no effect on morphine acute antinociception.

During the tolerance dosing regimen, chronic administration of morphine led to the induction of morphine tolerance as measured by the hotplate. Administration of O-1966 prior to each morphine injection during the chronic dosing regimen led to a significantly more pronounced tolerance than did morphine alone. Oppositely, when morphine was administered prior to O-1966 during the chronic dosing regimen, this combination led to the development of less tolerance than did chronic administration of morphine alone. Taken together, these results suggest that two distinct mechanisms of O-1966 action are mediating these opposing effects on the development of morphine antinociceptive tolerance.

These observations that pretreatment with O-1966 led to decreased morphine acute antinociception and increased morphine antinociceptive tolerance led us to speculate that O-1966 was directly affecting the function of the mu-opioid receptor, as it appears from these data that O-1966 is interfering with mu-opioid receptor activation acutely and mu-opioid receptor availability following the tolerance dosing regimen. We observed that O-1966 dose-dependently decreased [3H] DAMGO binding, but only at a high concentration, with a K_i_ value for O-1966 of 3.04 µM. These data suggest that O-1966 may be functioning as a negative allosteric modulator at the mu-opioid receptor, interfering with the orthostatic binding site. In the GTPγS binding assay, done in MOR-CHO cells absent of CB2 receptors, we found that administration of O-1966 decreased functional activation of the mu-opioid receptor by morphine. The observed interaction between O-1966 and functional activity was also shown to be dependent on order of administration, in that application of O-1966 prior to morphine decreased GTPγS-activation, while application of morphine followed by O-1966 did not impact the ability of morphine to stimulate the G-protein. The effect of O-1966 pretreatment on morphine-stimulated GTPγS binding was also blocked by co-administration with the CB2 receptor antagonist SR144528, as was seen on the hotplate, again suggesting that O-1966, and well as SR144528, interactions with morphine are mediated at least in part by direct activity on mu-opioid receptors. These data suggest that we observe different pharmacological effects of O-1966 on morphine antinociception and tolerance based on order of administration based on whether the presence of O-1966 is interfering with morphine binding at the mu-opioid receptor.

There are other examples in the literature of cannabinoid compounds that can interact in a similar manner with the mu-opioid receptor (see Raffa and Ward 2012 for review). For example, the phytocannabinoids THC and cannabidiol, which share several structural similarities with O-1966, have also been reported as allosteric modulators at the mu and delta opioid receptors (Kathmann et al., 2006). Additional reports have linked CB1 selective antagonists with direct actions on mu-opioid receptors. For example, the CB1 selective antagonist SR141716 (AKA rimonabant) also significantly decreases both basal and DAMGO-stimulated GTPγS binding in MOR-CHO membranes and in mouse cortex and binds directly to MORs with low micromolar affinity (Cinar and Szucs 2009). Also, Seely et al. (2012) reported that SR141716 and the structurally similar CB1 receptor antagonist AM-251 bind with mid-nanomolar affinity to human mu-opioid receptors, antagonize morphine–induced G-protein activation in MOR-CHO cells, and attenuate morphine antinociception.

Our results suggest that the presence of the CB2 agonist O-1966 may alter the functional activity of morphine at the receptor, impacting both the acute antinociceptive effects of morphine as well as its ability to produce antinociceptive tolerance. To follow up on this line of thinking, we tested the hypothesis that our findings were a result of O-1966-mediated disruption of mu-opioid receptor homodimerization that might lead to less analgesic efficacy but increased mu-opioid receptor internalization. Studies from a number of laboratories have supported the notion that the mu opioid receptor forms both homodimers and heterodimers with other class A GPCRs, and the functional activity of these oligomers is the subject of ongoing research (Ferre et al., 2014; Moller et al., 2020). We considered the possibility that the pre-treatment with O-1966 might alter the physical status of mu-opioid receptor homodimerization. Indeed, binding pockets have been identified, that when occupied, can impact mu-opioid receptor homodimerization (Zheng et al., 2012), so perhaps O-1966 binding was interrupting this process. Our FRET results showed that co-transfected cells treated with either morphine or O-1966, followed with either O-1966 or morphine, respectively, showed some reduction in the level of dimerization, based on inhibition of FRET. However, the data show that the pre-treatment with O-1966 did not significantly change the dimer status when compared to morphine pre-treatment. In no case was there a statistically significant difference between the morphine and O-1966 pretreatment groups. Taken together, the mechanism of the O-1966 effect on the function of MOR is not clear at this time, but suggest that O-1966 functions as a negative allosteric modulator at the mu-opioid receptor, leading to attenuation of the acute antinociceptive effects of morphine, but additional experiments are needed to determine this and rule out a role for direct activation of CB2 receptors on this interaction.

As mentioned previously, we did observe that when O-1966 treatment followed daily morphine administration, this combination lessened the development of antinociceptive tolerance and hyperalgesia. This supported our initial hypothesis, which we formed based on other work showing interplay between CB2 receptors, inflammation, and morphine tolerance (e.g. Huang et al., 2012; Jin et al., 2012; Jun et al., 2013; Vacca et al., 2013). We did not test whether our morphine/O-1966 dosing regimes altered inflammation in the present study, but as previously mentioned we have extensively characterized the protective and anti-inflammatory effects of the CB2 receptor agonist O-1966 in several rodent models of CNS injury (Zhang et al., 2007; Adhikary et al., 2011; Elliott et al., 2011; Amenta et al., 2012; Ramirez et al., 2012; Ronca et al., 2015). Therefore based on the order effects of our data results suggest that when O-1966 is administered following morphine (and mu receptor signalling is not impacted), O-1966 is working through a CB2 receptor mediated anti-inflammatory mechanism to decrease the development of morphine tolerance. As mentioned in the methods section, the affinity of O-1966 for CB1 and CB2 cannabinoid receptors was reported previously to be 5055 ± 984 and 23 ± 2.1 nmol/L, and we have not observed any hallmark CB1 receptor activation effects of O-1966 throughout our experience with the compound.

Further studies must be undertaken to determine whether this attenuation, as well as the attenuation observed in morphine hyperalgesia, was associated with anti-inflammatory, glial-inhibitory effects of O-1966 in this assay. Lastly, given the identification of sex differences regarding opioid analgesia and analgesic tolerance, cannabinoid pharmacology, as well as neuroinflammation, further work should also be conducted in female rodent models.

In conclusion, results from the present experiments provide surprising evidence of interactions between the CB2 receptor selective agonist O-1966 and morphine that are likely mediated in part by direct binding activity of O-1966 on the mu-opioid receptor, a property shared by other cannabinoid ligands as well. This interaction results in decreased potency of morphine to produce acute thermal antinociceptive effects but can also lead to the potentiation of morphine antinociceptive tolerance, suggesting complex alterations in morphine signaling. However, O-1966 co-administration also blocked morphine hyperalgesia, and led to an attenuation of morphine tolerance when administration followed each morphine injection, perhaps due to well-documented anti-inflammatory effects of CB2 receptor agonism. Overall, these data demonstrate that like other cannabinoid ligands, CB2 receptor ligands can influence the antinociceptive effects of morphine, and more work needs to be done to determine the clinical implications of these interactions, given the promise of CB2 receptor agonist pharmacotherapy for treatment of diseases and disorders associated with CNS injury that are often accompanied by opioid analgesia use.

## Data Availability

The raw data supporting the conclusions of this article will be made available by the authors, without undue reservation.
